# *Cryptosporidium baileyi* Pulmonary Infection in Immunocompetent Woman with Benign Neoplasm

**DOI:** 10.3201/eid2608.201117

**Published:** 2020-08

**Authors:** Żaneta Kopacz, Martin Kváč, Paweł Piesiak, Magdalena Szydłowicz, Andrzej B. Hendrich, Bohumil Sak, John McEvoy, Marta Kicia

**Affiliations:** Wroclaw Medical University, Wroclaw, Poland (Ż. Kopacz, P. Piesiak, M. Szydłowicz, A.B. Hendrich, M. Kicia);; Biology Centre of the Czech Academy of Sciences, České Budějovice, Czech Republic (M. Kváč, B. Sak);; University of South Bohemia, České Budějovice (M. Kváč);; North Dakota State University, Fargo, North Dakota, USA (J. McEvoy)

**Keywords:** parasites, parasitic diseases, protozoa, Cryptosporidium baileyi, pulmonary infection, benign neoplasm, immunocompetence, hamartoma, lung, zoonoses, Poland, enteric infections

## Abstract

*Cryptosporidium baileyi*, a bird-specific parasite, infects gastrointestinal, pulmonary, and urinary tracts of its host. We report on a *C. baileyi* infection associated with pulmonary hamartoma in an immunocompetent patient in Poland. Further work is needed to investigate the association between *Cryptosporidium* infections and tumors.

*Cryptosporidium* is a global protozoan parasite infecting wild, agricultural, and domestic vertebrates, and humans. Most infections lead to a gastrointestinal illness characterized by diarrhea (*1*). In addition, in birds, *C. baileyi* and *C. avium* parasites cause pulmonary infection (*2*). In humans, respiratory cryptosporidiosis is mostly associated with immunodeficiency, but several cases in immunocompetent children with intestinal cryptosporidiosis also have been reported (*3*,*4*). We describe a *C. baileyi* infection associated with a lung hamartoma in an immunocompetent patient.

The Human Research Ethics Committee of Wroclaw Medical University (Wroclaw, Poland) approved the use of diagnostic samples and corresponding patient data for this study (permit no. KB648/2014). The patient provided written informed consent for conduct of the studies and publication of the results.

In June 2015, a 51-year-old immunocompetent woman living in a rural area of Poland was admitted to a hospital with a suspected spinal injury. In addition to confirming the spinal injury, a solitary pulmonary nodule (SPN), 1.3–1.8 cm, in the third segment of the right lung upper lobe was detected by chest radiography and computed tomography (Figure, panels A, B). The lesion was of high density and had well-defined borders. The patient was referred to the Department of Pulmonology and Lung Cancers of Wroclaw Medical University for a nodule differential diagnosis. A bronchoscopy performed in July 2015 included biopsy samples for histology and cytology and a bronchial washings (BW) sample for acid-fast bacilli examination. We did not detect any changes in the bronchial trees, vocal cords, or trachea. Histologic and cytologic examination showed low cell count material, and the cells of the nodule lacked features of malignant process. The BW was negative for acid-fast bacilli. A second chest radiograph and bronchoscopy performed before surgery in September 2015 to remove the SPN showed a normal bronchial tree without anatomic changes and a slight increase in size of the SNP (1.5–2.0 cm). The SPN was removed by wedge resection in video-assisted thoracoscopic surgery after stabilization of the spine.

**Figure:**
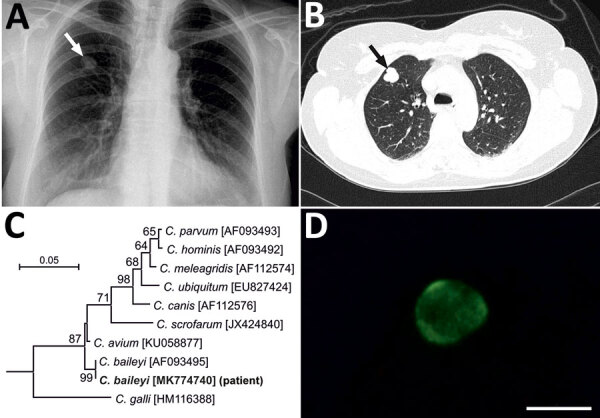
Findings from a 51-year-old immunocompetent woman with a benign neoplasm and *Cryptosporidium baileyi* pulmonary infection, Poland, 2015. A) Chest radiography in posterior-anterior position. A tumor, 13 × 18 mm with well-defined boundaries, is visible in the third segment of the upper right lung (arrow). B) Patient’s lung tomogram. Tumor is visible in the right lung (arrow). C) Maximum log likelihood tree based on partial sequences of gene coding small subunit rRNA of *Cryptosporidium*, including sequences obtained in this study (bold). Scale bar indicates nucleotide substitutions per site. D) *Cryptosporidium* oocyst detected in patient’s bronchial washings after immunofluorescent labeling with excitation and emission spectrum peak wave lengths of 495 nm/519 nm. Scale bar indicates 5 μm.

Before and during hospitalization, the patient was in good physical condition, afebrile, and without signs or symptoms of pulmonary and gastrointestinal infection. She reported no chronic diarrhea or cough in recent years. Blood count data, acid-base balance, gasometry, and spirometry did not deviate from mean values. She had no palpable lymphadenopathy. Except for the SPN, medical history was unremarkable. The patient was not undergoing immunosuppressive treatment and did not have an autoimmune disease. She was discharged 1 week after surgery in good general condition.

A paraffin-embedded tissue sample from the removed mass was diagnosed as a bronchial hamartoma. The BW was tested for pulmonary pathogens using individual conventional PCR (*Chlamydia pneumoniae*, *Mycoplasma pneumoniae*, *Legionella pneumophila*, *Cryptosporidium*, *Encephalitozoon*, and *Enterocytozoon*) and real-time PCR (*Pneumocystis jirovecii*). To identify *Cryptosporidium*, we extracted DNA from the BW and amplified a partial sequence of the small-subunit rRNA of *Cryptosporidium* (*5*) and sequenced it in triplicate. We inferred relationships to sequences from known species/genotypes from maximum-likelihood phylogenies (MEGA X, https://www.megasoftware.net). Bootstrap support for branching was based on 1,000 replications. A BW smear was labeled with genus-specific fluorescein isothiocyanate–conjugated antibodies (indirect fluorescent antibody Crypto cel; Cellabs Pty Ltd., https://www.cellabs.com.au).

The small-subunit sequence (GenBank accession no. MK774740) for the patient isolate was identical to that of a *C. baileyi* isolate from chickens (GenBank accession no. AF093495) (Figure, panel C). Microscopic examination of the BW smear showed oocysts measuring 5.2–5.5 μm with typical green fluorescence after labeling with FITC-conjugated anti-*Cryptosporidium* oocyst wall antibody (Figure, panel D).

We report an unusual case of human respiratory *C. baileyi* infection in an immunocompetent woman with hamartoma. Previously, a presumptive *C. baileyi* infection had been reported in the lungs, trachea, larynx, esophagus, whole intestine, gallbladder, and urinary tract of an immunodeficient man with HIV (*6*). Although not confirmed by sequence analysis, the finding that oocysts were infective for chickens supports the identification.

Immunocompetence is a critical determinant of the clinical course of *Cryptosporidium* infection. Intestinal cryptosporidiosis can be devastating to immunodeficient persons (*7*) and can involve extraintestinal sites (*6*). Pulmonary cryptosporidiosis in immunocompetent humans typically manifests as a cough and dyspnea, and concurrent intestinal infection is frequently reported (*3*,*4*). Consistent with this, the overwhelming majority of respiratory cases in humans have been caused by *C. parvum* and *C. hominis*, the major causes of intestinal cryptosporidiosis (*3*,*4*,*8*). Respiratory *Cryptosporidium* infection in birds is mostly asymptomatic but can result in high rates of death in association with other etiologic agents (*2*). The patient in this study did not report direct contact with birds but might have been exposed to wild bird excretions occupationally through work in an orchard. The hamartoma could have created an environment that supported the *C. baileyi* infection. *C. meleagridis* parasites were recently detected around a colon adenocarcinoma in an immunocompetent patient who, similarly, did not have symptoms typically associated with cryptosporidiosis (*9*). Alternatively, experimental evidence suggests that *Cryptosporidium* parasites can cause neoplastic changes in immunocompromised animals and in cells cultured in vitro, consistent with a role in carcinogenesis (*10*).
